# The Potential of Adding Mammography to Handheld Ultrasound or Automated Breast Ultrasound to Reduce Unnecessary Biopsies in BI-RADS Ultrasound Category 4a: A Multicenter Hospital-Based Study in China

**DOI:** 10.3390/curroncol30030251

**Published:** 2023-03-13

**Authors:** Wenhui Ren, Xuelian Zhao, Xiaowei Zhao, Huijiao Yan, Shangying Hu, Youlin Qiao, Zhijian Xu, Fanghui Zhao

**Affiliations:** 1 Department of Cancer Epidemiology, National Cancer Center/National Clinical Research Center for Cancer/Cancer Hospital, Chinese Academy of Medical Sciences and Peking Union Medical College, Beijing 100021, China; 2 Department of Cancer Prevention, National Cancer Center/National Clinical Research Center for Cancer/Cancer Hospital, Chinese Academy of Medical Sciences and Peking Union Medical College, Beijing 100021, China; 3 Center for Global Health, School of Population Medicine and Public Health, Chinese Academy of Medical Sciences & Peking Union Medical College, Beijing 100730, China

**Keywords:** breast neoplasms, ultrasonography, automated breast ultrasound, mammography, diagnosis

## Abstract

The appropriate management strategies for BI-RADS category 4a lesions among handheld ultrasound (HHUS) remain a matter of debate. We aimed to explore the role of automated breast ultrasound (ABUS) or the second-look mammography (MAM) adjunct to ultrasound (US) of 4a masses to reduce unnecessary biopsies. Women aged 30 to 69 underwent HHUS and ABUS from 2016 to 2017 at five high-level hospitals in China, with those aged 40 or older also accepting MAM. Logistic regression analysis assessed image variables correlated with false-positive lesions in US category 4a. Unnecessary biopsies, invasive cancer (IC) yields, and diagnostic performance among different biopsy thresholds were compared. A total of 1946 women (44.9 ± 9.8 years) were eligible for analysis. The false-positive rate of category 4a in ABUS was almost 65.81% (77/117), which was similar to HHUS (67.55%; 127/188). Orientation, architectural distortion, and duct change were independent factors associated with the false-positive lesions in 4a of HHUS, whereas postmenopausal, calcification, and architectural distortion were significant features of ABUS (all *p* < 0.05). For HHUS, both unnecessary biopsy rate and IC yields were significantly reduced when changing biopsy thresholds by adding MAM for US 4a in the total population (scenario #1:BI-RADS 3, 4, and 5; scenario #2: BI-RADS 4 and 5) compared with the current scenario (all *p* < 0.05). Notably, scenario #1 reduced false-positive biopsies without affecting IC yields when compared to the current scenario for ABUS (*p* < 0.001; *p* = 0.125). The higher unnecessary biopsy rate of category 4a by ABUS was similar to HHUS. However, the second-look MAM adjunct to ABUS has the potential to safely reduce false-positive biopsies compared with HHUS.

## 1. Introduction

Mammography (MAM) is widely used as the standard modality for detecting and screening early breast cancer. However, the diagnostic accuracy of MAM is limited in women with dense breasts [1,2,3]. Another barrier for MAM to apply and expand sustainability is the lack of equipment, especially in low resources areas [4].

Conventional handheld ultrasound (HHUS) offers a low-cost and portable way of breast cancer detection without the limitations of breast density [5], thereby increasingly being used in clinical breast examination. However, operator dependence has long been a concern for HHUS and causes interobserver variability. Automated breast ultrasound (ABUS) is a newly designed tool with the potential to overcome the criticism of HHUS by separating image acquisition from interpretation to increase reproducibility [6]. Multiplanar reconstructions also provide an advantage for evaluating breast lesions which might help improve the diagnosis accuracy [6]. 

To provide standardized ultrasound (US) findings reporting systems and aid quality assurance and risk assessment, the Breast Imaging Reporting and Data System (BI-RADS) is generalized worldwide [7]. The latest nationwide survey in mainland China reported the average utilization rate of BI-RADS was up to 87.02% among 5460 departments providing ultrasound diagnoses [8]. However, the application of category 4 subdivisions in the new fifth BI-RADS lexicon offers a challenge for managing BI-RADS 4a. The malignant rate of BI-RADS category 4a is meager (2–10%), in which immediate biopsy referral is recommended, while BI-RADS category 3 refers to probably benign masses (<2%) with short-term follow-up imaging recommended [7]. In case to avoid missed diagnoses, observers tend to upgrade breast masses into 4a when it is difficult to determine category 3 or 4a, but this may result in unnecessary biopsies.

The benign biopsy rate on breast US of 4a patients is a considerable percentage (more than 50%) [9,10]. Unnecessary biopsies can result in negative consequences for normal women, including the risk of complications, psychological anxiety, and additional financial costs [11,12,13]. Previous studies about stratifying and managing the way of 4a patients were mainly focused on incorporating elastography into US workflows or developing predictive models including radionics and clinical factors [9,10,14,15]. However, avoiding excessive biopsies of HHUS category 4a by supplementing other techniques remains for further exploration.

The diagnostic performance between ABUS and HHUS has been proven comparable based on the fifth BI-RADS edition [16]. However, to our knowledge, there is not yet established evidence to identify whether the accuracy of category 4a on ABUS is higher than HHUS. Furthermore, given the advantages in diagnosing calcification lesions [17], MAM provides a potential complementary option to improve diagnostic performance when combined with US (ABUS or HHUS). Few studies have evaluated whether adding MAM to lesions assessed as US category 4a improves diagnostic accuracy and reduces unnecessary biopsies rate.

Therefore, we aimed to exploratorily assess the diagnostic performance of ABUS or the second-look MAM adjunct to US to help reduce false-positive diagnoses of 4a patients without impacting the breast cancer detection rate.

## 2. Materials and Methods

### 2.1. Study Population and Design

The research design has been published in detail elsewhere [18]. Briefly, this multicenter cross-sectional study was conducted in five high-level hospitals located in China (including Beijing, Tianjin, Shanghai, Hangzhou, and Guangzhou) from February 2016 to March 2017. Female outpatients with breast-related complaints were recruited in this study. The exclusion criteria included aged <30 and ≥70 years; previously received a diagnosis of or treatment for breast cancer; undergone surgical or percutaneous breast procedures in the past 12 months; had a history of lumpectomy, contra-lateral mastectomy, or breast augmentation; and currently pregnant, breastfeeding, or planning to become pregnant.

**Figure 1:**
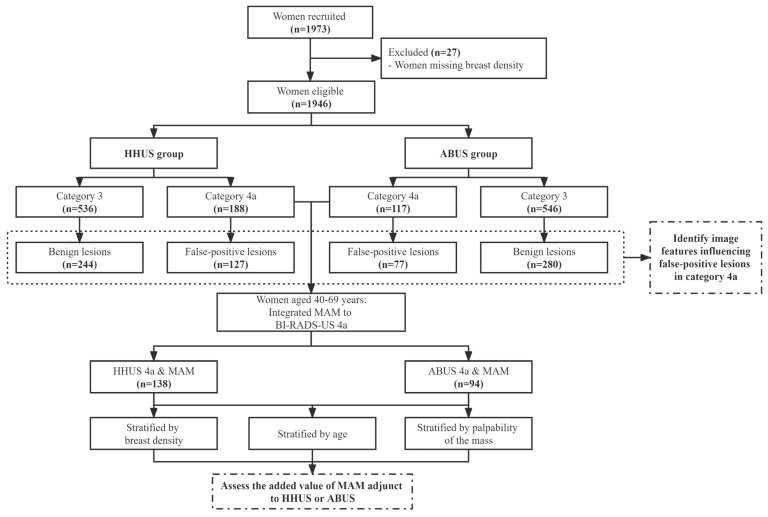
Flow chart of the study design. Abbreviations: ABUS: automated breast ultrasound; BI-RADS: Breast Imaging Reporting and Data System; HHUS: handheld ultrasound; MAM: mammography; US: ultrasound.

All participants were invited to attend both HHUS and ABUS, while those aged 40 years and above also underwent MAM. Patients with the most severe category on three modalities, including BI-RADS 4 and 5, were considered positive findings and required a biopsy, whereas those with BI-RADS 1, 2, or 3 were categorized as negative findings. The study was registered in the Chinese Clinical Trial Registry (ChiCTR1800017908) and approved by the Institutional Review Board of Cancer Institute, Chinese Academy of Medical Sciences (IRB approval No.15-061/988), and the Institutional Review Board of all participating hospitals. According to the study aims, we included the participants with HHUS or ABUS categories 3 and 4a as the analysis set. The study design was shown in Figure 1 in detail.

### 2.2. Image Acquisition and Interpretation

The participants underwent ABUS using Invenia ABUS (GE Healthcare, Sunnyvale, CA, USA) performed by technicians who received training for 3 days and interpreted by radiologists with 3–6 months of experience with ABUS. Three planes (including lateral, anteroposterior, and medial) are collected on each breast. The image in three views could be transmitted to the workstation and reconstructed in the breast and displayed in 3D volumes. The HHUS images were acquired by one of the following devices, including GE LOGIQ9 (GE Medical Systems, Milwaukee, WI, USA), iU22 Ultrasound System (Philips Medical System, Bothell, WA, USA), S2000 (Siemens Medical Solutions, Mountain view, CA, USA), and the Aixplorer system (Supersonic Imagine, Aix en Provence, France), which was performed by qualified radiologists with 5–25 years of experience in five hospitals. All MAM examinations were performed by one of three techniques including GE Sengraphe DS (GE Medical Systems, Milwaukee, WI, USA), Hologic Selenia (Hologic, Bedford, MA, USA), and Fujifilm FDR MS-2500 (Fujifilm Crop, Tokyo, Japan) and interpreted by doctors with 5–25 years of experience. All screening physicians involved in the study were trained in the protocol and related technical specifications and diagnostics before starting the study.

During the study, different experienced radiologists reviewed and interpreted images from three modalities and were blinded to each other. However, they were provided with information on participants’ clinical examinations. 

### 2.3. Statistical Analysis

We analyzed and compared the detection rate of normal/benign and malignant lesions classified as US categories 3, 4a, 4b, and 4c using the Chi-squared test for trend. In clinical practice, observers always have difficulty in better characterizing category 3 and 4a lesions even for highly qualified experts. Therefore, to evaluate the clinical and image features influencing the false-positive lesions in category 4a, we selected those who were categorized as 3 and 4a and underwent biopsy and evaluated them as benign breast lesions as an analysis set. With category 3 as the reference group, multivariable logistic regression analysis was used to estimate odds ratios (ORs) and confidence intervals (CIs). As for HHUS, the following characteristics of the lump were included in the analysis: maximum diameters, shape, orientation, margin, posterior feature, calcification, distorted structure, duct change, and vascularity. As for ABUS, we also analyzed retraction phenomenon in the coronal view. Furthermore, age, menopausal status, breast density, and palpability of the mass were also included in logistic regression analysis to control for potential confounding variables. Variables that were statistically significant in the univariate analysis would be prioritized for inclusion in the multivariate analysis, and for that were non-significant in the univariate but clinically valuable also be considered for analysis.

Unnecessary biopsy rate, invasive cancer (IC) detection rate, malignant rate of biopsy, sensitivity, specificity, positive predictive value (PPV), negative predictive value (NPV), and area under curve (AUC) were calculated to evaluate the diagnostic performance among different biopsy thresholds, which were compared using the McNemar’s tests or the Chi-squared test. The statistical analysis was performed with SAS, version 9.4 (SAS Institute, Cary, North Carolina). A *p*-value <0.05 was considered statistical significance.

## 3. Results

### 3.1. Distribution of Benign and Malignant Lesions According to BI-RADS-US Category

Among 1973 eligible women who received HHUS and ABUS between 2016 and 2017, 27 women were excluded for missing breast density in those who underwent MAM (Figure 1). Of 1946 participants (mean age 44.9 ± 9.8 years) for analysis, 188 (9.66%) were categorized as category 4a in HHUS while 117 (6.01%) of ABUS. For HHUS, the false-positive biopsy rate showed a decreasing trend among 4a (67.55%), 4b (26.39%), and 4c (18.99%) (*p* for trend <0.001). ABUS showed the same trend as HHUS among 4a, 4b, and 4c (65.81% vs. 23.94% vs. 8.57%; *p* for trend <0.001). Meanwhile, 72.84% of unnecessary biopsies occurred in 81 participants who have assessed the BI-RADS 4a category with both ABUS and HHUS (Table 1). 

### 3.2. Clinical and Imaging Factors Associated with False-Positive Lesions in Category 4a

Among 371 benign lesions assessed as categories 3 and 4a on HHUS, 127 were assessed as false-positive cases in 4a. Meanwhile, the false-positive cases were 77 in ABUS 4a among 357 benign lesions in 3 and 4a. Table 2 and Table 3 display the ORs of clinical and imaging factors for false-positive cases assigned by HHUS and ABUS when using category 3 as the reference group. In the logistic regression analysis, nonparallel masses (OR, 5.30; 95% CI, 1.98 to 14.16; *p* = 0.001), architectural distortion (2.86; 1.33 to 6.15; *p* = 0.007), and duct change (8.92; 3.49 to 22.77; *p* < 0.001) were independent factors linked with the false-positive lesions in the BI-RADS-US 4A in HHUS, while postmenopausal (0.37; 0.19 to 0.74; *p* = 0.005), calcification (2.27; 1.11 to 4.62; *p* = 0.024), and architectural distortion (4.05; 1.44 to 11.44; *p* = 0.008) were the significant features of ABUS.

### 3.3. Diagnostic Performance of Adding MAM to HHUS or ABUS

We evaluated the effect of changing biopsy thresholds for women with US category 4a lesions who underwent MAM among women aged 40 and above (HHUS, 138 women; ABUS, 94 women). Three scenarios about different biopsy thresholds are shown in Table 4, including all women with BI-RADS-US (HHUS or ABUS) category 4a undergoing biopsy (current scenario), women with BI-RADS-US category 4a and BI-RADS-MAM category 3, 4, and 5 undergoing biopsy (scenario #1), and women with BI-RADS-US category 4a and BI-RADS-MAM category 4 and 5 undergoing biopsy (scenario #2).

The diagnostic performance of different scenarios was compared among women with BI-RADS-US category 3 and 4a lesions (Table 4). The AUCs of the combination of HHUS and MAM (both scenarios #1 and #2) were similar to that of the current scenario (*p* = 0.238; *p* = 0.095). Meanwhile, only scenario #1, which adds MAM to ABUS, obtained a similar AUC compared with the current scenario (*p* = 0.277). Although sensitivity was significantly lower in both new scenario groups than in the current scenario group, specificity and PPV improved for HHUS and ABUS (all *p* < 0.05).

### 3.4. Value of Adding MAM to HHUS or ABUS in Reducing Unnecessary Biopsy

Table 5 shows the effect of increasing biopsy thresholds on unnecessary biopsy rate, IC detection rate, and malignancy rate of biopsy when integrating MAM with HHUS or ABUS. For HHUS, the unnecessary biopsy rate was significantly reduced to 39.86% (55/138) and 28.26% (39/138) for scenario #1 and scenario #2 compared with the current scenario, respectively (all *p* < 0.001), and the malignancy rate of biopsy increased to 45.54% (46/101) and 51.25% (41/80), respectively (*p* = 0.102; *p* = 0.008). However, both new scenarios had significantly lower IC detection rates than the current scenario (all *p* < 0.001). Similar patterns were recorded for ABUS, apart from scenario #1, which significantly reduced the false positive biopsies (*p* < 0.001) without decreasing IC yield (*p* = 0.125).

We also compared the unnecessary biopsy rates, IC yields, and malignant rate of biopsy between the two new scenarios and the current scenario by age, breast density, and palpability of the mass (Table 5). In all subgroups, a lower unnecessary biopsy rate was always significantly noted for the two new scenarios in both HHUS and ABUS (all *p* < 0.001). The IC yields of the two new biopsy thresholds were not inferior to the current scenario for HHUS in women with less dense breasts (*p* = 1.000) and those with palpable masses (*p* = 0.063). For ABUS, we did not observe a significant difference in diagnostic performance in all subgroups between the two new scenarios and the current scenario, except for the IC yields of scenario #2 of women with dense breasts (*p* = 0.016).

## 4. Discussion

The potentially large number of unnecessary biopsies resulting from the current recommendation for BI-RADS-US category 4a creates an additional burden for women and impacts clinical resources. Our findings showed that the false-positive rate of category 4a in ABUS was almost 65.81%, which was similar to HHUS (67.55%). Meanwhile, clinical and sonographic factors influencing the 4a false-positive lesions were observed differently between HHUS and ABUS, which might be associated with radiologists’ experiences and equipment difference. To note, the potential added value of the second-look MAM adjunct to HHUS 4a was identified to reduce unnecessary biopsy procedures among women with dense breasts or palpability masses without influencing the invasive cancer detection. In addition, the new strategy combining ABUS category 4a and MAM 3, 4, and 5 as a new biopsy threshold would have the potential to safely reduce false-positive biopsies.

Current criticisms of HHUS include concern about the false positive results and associated unnecessary biopsies [19]. The range of the malignancy rate for BI-RADS-US 4 lesions is wide (2~95%) [7]. In particular, considerable overlapped image features between benign and malignant lesions in category 4a result in difficulty to distinguish malignancy. The primary reason is lacking objective criteria for the subclassification of category 4 lesions which are largely based on the experience of the sonographers [20]. Our results also reflected that the benign biopsy rate of 4a was higher even when performed by highly qualified experts from high-level hospitals, which was following the conclusions of previous studies [9,10]. 

The potential of ABUS in the diagnostic setting of breast cancer has currently become the research focus because of its benefits [21]. Some unique features through multiplanar reconstructions can provide additional information for differentiating benign and malignant masses [22]. For example, the retraction phenomenon, as the specific feature observed in ABUS coronal view, has been suggested to be a predictable characteristic of breast cancer [23]. Our previous studies have suggested that specificity and PPV were significantly higher in ABUS, compared with that of HHUS [24,25]. However, this study showed that the unnecessary biopsy rate of ABUS among 4a masses is similar to that of HHUS. This might be explained by the lower ability of radiologists who review the ABUS images to evaluate category 4a even with standardized training before. Additionally, there is not yet a well-established specific criterion in determining lesion characteristics with ABUS images worldwide, primarily based on BI-RADS-US descriptors. Of note, the non-essential biopsy rate was 72.84% in 81 patients assessed with both ABUS and HHUS. Thereby, the technological inherent limitations of US equipment may also be another important reason.

In routine clinical practice, the interpretation criterion of category 4a is a mass with benign ultrasound appearance but exhibiting any suspicious sign [26]. Of all benign lesions, duct change, nonparallel masses, and architectural distortion increased the level of suspicion for these masses and preferred BI-RADS 4a to 3 for HHUS in our study. Surrounding background tissue change results in the poor demarcation between masses and normal tissue, which may partly be explained by these features impeding the evaluation accuracy the breast lesion [27,28,29]. Meanwhile, calcification was observed as being associated with false-positive cases in lesions of category 4a examined using ABUS. Due to the influence of probe frequency, tissue background echo, and operator technology, US is not ideal to detect microcalcification in lesions even though it is the key imaging feature for the diagnosis of breast cancer [30]. Notably, we also found that menopausal status tended to have higher probability of false positives. ABUS separates image acquisition (performed by the technicians) from interpretation. Therefore, sonographers will pay more attention to the clinical characteristics of patients compared with HHUS, such as menopausal status. Above all, benign possibilities should be taken into account when these features are found, which suggests that examiners need to integrate other important image features when interpreting ultrasound images by receiving specific training about BI-RADS descriptors. More importantly, supplemental other diagnostic tools might be effective strategies to help triage populations with lower risk by delaying biopsy interventions and avoiding making unnecessary recommendations.

Previous works have explored the management strategies of US category 4a. Several studies mainly focused on the new US imaging technique, elastography, and have confirmed the potential of combined shear wave and strain elastography to US to reduce unnecessary biopsies in breast cancer diagnostics [10,14,15]. However, the evidence of evaluating the value of other methods added to US is scant. To date, no other trials of integrated MAM in US have reported results. Lacking sufficient evidence to reduce breast cancer mortality could be a barrier to implementing the widespread US as the stand-alone screening modality. Currently, supplemental US to MAM has become a mainstay of diagnostic breast imaging for women with mammographically dense breasts. Some low- and middle-income countries (LMICs) are exploring HHUS application as a primary screening method for breast cancer because of the advantages of being cheap, having higher access, and being noninvasive [31,32,33]. A systematic review demonstrated that studies focusing on HHUS applications in LMICs have risen nearly by 60%, which reveals the increasing adoption of HHUS equipment worldwide [34]. However, given the lower specificity and higher false-positive rate of HHUS, it is important to explore US-based diagnostic strategies in combination with other techniques. 

US (HHUS or ABUS) category 4a combined with MAM positive results (category 4 and above) as the biopsy threshold can significantly improve diagnostic performance and reduce false-positive biopsies when compared to the current scenario, but probably with the risk of missing invasive cancer. The most likely explanation is that more than 70% of participants were aged 40 and older and almost 50% of them were premenopausal who underwent MAM and were found to have dense breasts in our study, which may be associated with the lower sensitivity of MAM [18]. Of note, our findings also revealed that the new biopsy threshold did not affect the invasive cancer yield, which is comparative with the current scenario for women with less dense breasts. Furthermore, this study was conducted in hospitals and the conclusions came from the symptomatic population who has a higher risk for breast cancer than the asymptomatic population. In view of these issues, whether an immediate biopsy strategy is needed for this group still depends on clinicians’ perceptions of acceptable risks based on an individual patient basis to balance the pros and cons. 

Notably, we found that the added value of the second-look MAM adjunct to HHUS 4a could acquire higher cancer yields when breast masses were palpable, which might be related to the probability of malignancy being fairly high in palpable lesions. Palpability is likely to be viewed with more suspicion by these masses, providing information to aid diagnosis for radiologists [35]. A previous study showed the combination of MAM and HHUS could potentially increase the negative predictive value among women with palpable breast abnormality [36]. 

Most importantly, this study provides a more practical perspective that when the biopsy threshold identified BI-RADS 3 and above for MAM combined with BI-RADS 4a for ABUS has benefited over the current biopsy strategy for reducing false-positive biopsies without affecting the detection performance. Findings from a prospective study indicated that ABUS has a higher ability to detect architectural distortions, one of the risk factors of subsequent breast cancer in mammographic findings [37], on the coronal plane than HHUS [38]. Additionally, ABUS can supplement mammography to detect more non-calcified carcinomas compare with HHUS in women with dense breasts [38]. This might explain the higher diagnostic performance of the biopsy threshold (scenario #2) for ABUS than that of HHUS. Furthermore, we also acknowledged that the difference between HHUS and ABUS might result from the limited sample size in category 4a. 

The reasons for false-positive findings need to be identified through external quality assessment in clinical practice. Some cases without abnormal pathological findings might have image changes that mimic the appearance of precancerous lesions, resulting in misclassification as positive results. This group then needs to be given priority attention, because the image feature abnormalities are more likely to be risk markers of breast cancer [39]. A previous retrospective study performed by Hofvind et al. showed that a higher interval breast cancer rate appeared after a false-positive result in a MAM-based screening program [40]. The biological susceptibility maybe contributes to the increased risk for breast cancer [41]. Thereby, risk-based stratification management strategies play a vital role for women with false-positive results. However, because of our cross-sectional study design, future works should be conducted to explore the safe screening intervals for false-positive recalls.

The main strength of this study is that it is the first to evaluate the added value of the second-look MAM adjunct to US (HHUS or ABUS) category 4a. It possibly contributes to the understanding that MAM might be a useful additional tool for US in breast cancer diagnostics to better distinguish which patients require a histopathologic confirmation of suspicious lesions on imaging. This also provides potentially helpful strategies for improving diagnostic performance in areas where US is applied as the first-line breast diagnostic method.

This study had several limitations. First, the experience of the radiologists among five research centers could affect the ability of image acquisition and interpretation. However, the variability among radiologists might be avoided to some extent by the standardized training before the research. Another limitation is that the absence of follow-up information may affect the accurate evaluation of long-term effectiveness results in patients with false-positive biopsies of US 4a among different biopsy thresholds. In addition, the study participants were recruited from hospital outpatients with a higher risk of breast cancer, which does not reflect the new biopsy thresholds applications for the general population. To address this issue, we now have conducted ongoing real-world research to explore the screening effectiveness for HHUS, ABUS, and MAM in average-risk populations.

## 5. Conclusions

The higher unnecessary biopsy rate of category 4a by ABUS was very similar to HHUS, reflecting the image factors influencing the false positive 4a lesions should be the focus of integrated training. The second-look MAM adjunct to HHUS had the potential to reduce overdiagnosis for women with less dense breasts or palpable breast masses. Notably, BI-RADS 3 and above for MAM combined with BI-RADS 4a for ABUS benefited from the current biopsy strategy and safely reduced false-positive biopsies. Future work is still needed to explore the appropriate follow-up interval for false-positive patients in specific populations.

## Figures and Tables

**Table 1:** Distribution of benign and malignant lesions according to BI-RADS-US category between HHUS and ABUS.

BI-RADS US Category	Total (N, %) *	Normal/Benign (n, %)	DCIS (n, %)	IC (n, %)
**HHUS**				
3	536 (27.54)	518 (96.64)	9 (1.68)	9 (1.68)
4a	188 (9.66)	127 (67.55)	10 (5.32)	51 (27.13)
4b	72 (3.70)	19 (26.39)	11 (15.28)	42 (58.33)
4c	79 (4.05)	15 (18.99)	6 (7.59)	58 (73.42)
*p* for trend	-	<0.001	-	<0.001
**ABUS**				
3	546 (28.06)	520 (95.24)	5 (0.91)	21 (3.85)
4a	117 (6.01)	77 (65.81)	12 (10.26)	28 (23.93)
4b	71 (3.65)	17 (23.94)	10 (14.09)	44 (61.97)
4c	105 (5.40)	9 (8.57)	9 (8.57)	87 (82.86)
*p* for trend	-	<0.001	-	<0.001
**HHUS & ABUS**				
3	436 (22.40)	424 (97.25)	4 (0.92)	8 (1.83)
4a	81 (4.16)	59 (72.84)	6 (7.41)	16 (19.75)
4b	21 (1.08)	5 (23.81)	3 (14.29)	13 (61.90)
4c	32 (1.64)	2 (6.25)	3 (9.37)	27 (84.38)
*p* for trend	-	<0.001	-	<0.001

* The denominator of the percentage of BI-RADS categories 3 and 4 is 1946. Abbreviations: ABUS: automated breast ultrasound; BI-RADS: Breast Imaging Reporting and Data System; DCIS: ductal carcinoma in situ; HHUS: handheld ultrasound; IC: invasive cancer.

**Table 2:** Differential regression analysis of clinical and imaging features of false-positive lesions in BI-RADS 4a among HHUS.

Variables	BI-RADS 4a (Benign, n = 127)	BI-RADS 3 (Benign, n = 244)	OR (95% CI)	aOR (95% CI) **
**Age** (**y**)				
30–39	48	102	1.00	
40–69	79	142	1.18 (0.76, 1.84)	-
**Menopausal status**				
Premenopausal	25	44	1.00	
Postmenopausal	102	200	0.90 (0.52, 1.55)	-
Breast density *				
Less dense	11	21	1.00	
More dense	68	121	1.17 (0.76, 1.80)	-
**Palpability of the mass**				
Palpable	62	77	1.00	1.00
Non palpable	65	147	0.69 (0.45, 1.07)	0.84 (0.48, 1.49)
**Size** (**cm**) *				
≤2	79	184	1.00	1.00
>2	43	60	1.57 (0.98, 2.51)	1.61 (0.87, 2.97)
Shape *				
Oval and Round	56	174	1.00	1.00
Irregular	66	70	2.69 (1.72, 4.20)	1.69 (0.95, 3.03)
Orientation *				
Parallel	102	236	1.00	1.00
Nonparallel	20	8	5.51 (2.35, 12.92)	5.30 (1.98, 14.16)
Margin *				
Regular	74	204	1.00	1.00
Irregular	48	40	3.10 (1.89, 5.07)	1.68 (0.88, 3.20)
Posterior feature *				
None	87	182	1.00	
Enhancement and/or Shadowing	35	62	1.12 (0.69, 1.81)	-
Calcification *				
None	97	213	1.00	1.00
Present	25	31	1.68 (0.95, 3.00)	1.82 (0.91, 3.61)
**Distorted structure**				
None	102	227	1.00	1.00
Architectural distortion	25	17	3.27 (1.69, 6.33)	2.86 (1.33, 6.15)
**Duct change**				
None	102	236	1.00	1.00
Dilation or with filling	25	8	7.23 (3.16, 16.57)	8.92 (3.49, 22.77)
**Vascularity**				
Absent	70	171	1.00	1.00
Internal and/or vessels vascularity	57	73	1.91 (1.22, 2.97)	1.24 (0.71, 2.16)

* missing values in data; ** OR was adjusted by the following variables: palpability of the mass, size, shape, orientation, margin, calcification, distorted structure, duct change, and vascularity. Abbreviations: BI-RADS: Breast Imaging Reporting and Data System; HHUS: handheld ultrasound.

**Table 3:** Differential regression analysis of clinical and imaging features of false-positive lesions in BI-RADS 4a among ABUS.

Variables	BI-RADS 4a (Benign, n = 77)	BI-RADS 3 (Benign, n = 280)	OR (95% CI)	aOR (95% CI) **
**Age** (**y**)				
30–39	26	119	1.00	
40–69	51	161	1.45 (0.86, 2.46)	-
**Menopausal status**				
Premenopausal	24	48	1.00	1.00
Postmenopausal	53	232	0.46 (0.26, 0.81)	0.37 (0.19, 0.74)
Breast density *				
Less dense	10	25	1.00	
More dense	45	136	1.21 (0.73, 2.00)	-
**Palpability of the mass**				
Palpable	38	122	1.00	1.00
Non palpable	39	158	0.79 (0.48, 1.31)	0.78 (0.41, 1.48)
**Size** (**cm**) *				
≤2	48	215	1.00	1.00
>2	23	56	1.70 (0.96, 3.01)	1.90 (0.91, 3.97)
Shape *				
Oval and Round	35	201	1.00	1.00
Irregular	36	70	2.63 (1.56, 4.44)	2.23 (0.99, 4.99)
Orientation *				
Parallel	57	242	1.00	1.00
Nonparallel	14	29	1.92 (0.96, 3.85)	1.42 (0.56, 3.56)
Margin *				
Regular	30	177	1.00	1.00
Irregular	41	94	2.25 (1.35, 3.76)	0.96 (0.44, 2.11)
**Posterior feature**				
None	44	183	1.00	
Enhancement and/or Shadowing	33	97	1.42 (0.85, 2.37)	-
**Calcification**				
None	52	243	1.00	1.00
Present	25	37	3.16 (1.75, 5.69)	2.27 (1.11, 4.62)
**Distorted structure**				
None	62	270	1.00	1.00
Architectural distortion	15	10	6.53 (2.80, 15.22)	4.05 (1.44, 11.44)
**Duct change**				
None	62	257	1.00	1.00
Dilation or with filling	15	23	2.70 (1.33, 5.48)	2.20 (0.90, 5.39)
**Retraction phenomenon**				
None	71	280		
Present	6	0	-	-

* missing values in data; ** OR was adjusted by the following variables: menopausal status, palpability of the mass, size, shape, orientation, margin, calcification, distorted structure, and duct change. Abbreviations: ABUS: automated breast ultrasound; BI-RADS: Breast Imaging Reporting and Data System.

**Table 4:** Diagnostic performance of different biopsy thresholds when adding MAM to HHUS or ABUS.

Biopsy Thresholds	HHUS + MAM (N = 138)					ABUS + MAM (N = 94)				
	Sensitivity (%, 95% CI)	Specificity (%, 95% CI)	PPV (%, 95% CI)	NPV (%, 95% CI)	AUC Value (95% CI)	Sensitivity (%, 95% CI)	Specificity (%, 95% CI)	PPV (%, 95% CI)	NPV (%, 95% CI)	AUC Value (95% CI)
Current scenario	77.22 (66.14, 85.60)	80.31 (76.98, 83.27)	32.45 (25.92, 39.71)	96.64 (94.64, 97.64)	0.80 (0.75, 0.85)	60.61 (47.80, 72.18)	87.10 (84.08, 89.63)	34.19 (25.83, 43.60)	95.24 (93.01, 96.81)	0.77 (0.70, 0.84)
Scenario #1	59.49 (47.84, 70.21)	91.01 (88.47, 93.05)	44.76 (35.15, 54.76)	94.83 (92.70, 96.38)	0.78 (0.72, 0.84)	50.00 (37.56, 62.44)	94.30 (92.05, 95.96)	49.25 (36.95, 61.64)	94.46 (92.23, 96.10)	0.74 (0.68, 0.81)
Scenario #2	51.90 (40.44, 63.17)	93.95 (91.75, 95.61)	51.25 (39.89, 62.48)	94.10 (91.92, 95.74)	0.76 (0.70, 0.82)	40.91 (29.18, 53.70)	95.64 (93.59, 97.08)	50.94 (37.00, 64.75)	93.61 (91.29, 95.36)	0.70 (0.63, 0.76)
* p value_1_	<0.001	<0.001	0.036	0.131	0.238	0.016	<0.001	0.044	0.555	0.277
** *p* value_2_	<0.001	<0.001	0.004	0.041	0.095	<0.001	<0.001	0.038	0.229	0.018

The diagnostic performance of different scenarios was compared among women with BI-RADS-US (HHUS or ABUS) category 3 and 4a lesions. Current scenario: all women with BI-RADS-US (HHUS or ABUS) category 4a underwent biopsy; scenario #1: women with BI-RADS-US category 4a and BI-RADS-MAM category 3, 4, and 5 underwent biopsy; scenario #2: women with BI-RADS-US category 4a and BI-RADS-MAM category 4 and 5 underwent biopsy. * compare scenario #1 with the current scenario; ** compare scenario #2 with the current scenario. Abbreviations: ABUS: automated breast ultrasound; HHUS: handheld ultrasound; MAM: mammography; PPV: positive predictive value; NPV: negative predictive value; AUC: area under curve.

**Table 5:** Effect of increasing biopsy thresholds on unnecessary biopsies and cancer yields when adding MAM to HHUS or ABUS.

Biopsy Thresholds	HHUS + MAM (N = 138)			ABUS + MAM (N = 94)		
	Unnecessary Biopsy Rate (n, %)	IC Detection Rate (n, %)	Malignancy Rate of Biopsy (n, %)	Unnecessary Biopsy Rate(n, %)	IC Detection Rate (n, %)	Malignancy Rate of Biopsy (n, %)
**Total**						
Current scenario	84 (60.87)	46 (33.33)	54 (39.13)	55 (58.51)	28 (29.78)	39 (41.49)
Scenario #1	55 (39.86) *	38 (27.54) †	46 (45.54)	33 (35.11) *	24 (25.53)	33 (50.00)
Scenario #2	39 (28.26) *	34 (24.64) †	41 (51.25)	26 (27.66) *	20 (21.28) †	27 (50.94)
**Stratified by breast density**						
**Less dense**						
Current scenario	11 (52.38)	8 (38.10)	10 (47.62)	10 (55.56)	4 (22.22)	8 (44.44)
Scenario #1	8 (38.10)	8 (38.10)	10 (55.56)	4 (22.22) *	4 (22.22)	6 (60.00)
Scenario #2	5 (23.81) *	7 (33.33)	9 (64.29)	3 (16.67) *	3 (16.67)	5 (62.50)
**More dense**						
Current scenario	73 (72.39)	38 (32.48)	44 (37.61)	45 (59.21)	24 (31.58)	31 (40.79)
Scenario #1	47 (40.17) *	30 (25.64) †	36 (43.37)	29 (38.16) *	20 (26.32)	27 (48.21)
Scenario #2	34 (29.06) *	27 (23.08) †	32 (48.48)	23 (30.26) *	17 (22.37) †	22 (48.89)
**Stratified by age**						
**40–49 years**						
Current scenario	52 (73.24)	17 (23.94)	19 (26.76)	34 (68.00)	14 (28.00)	16 (32.00)
Scenario #1	34 (47.89) *	13 (18.31)	15 (30.61)	22 (44.00) *	12 (24.00)	14 (38.89)
Scenario #2	29 (40.85) *	11 (15.49) †	13 (30.95)	19 (38.00) *	10 (20.00)	12 (38.71)
**50–69 years**						
Current scenario	32 (47.76)	29 (43.28)	35 (52.24)	21 (47.72)	14 (31.81)	23 (52.27)
Scenario #1	21 (31.34) *	25 (37.31)	31 (59.62)	11 (25.00) *	12 (27.27)	19 (63.33)
Scenario #2	10 (14.93) *	23 (34.33) †	28 (73.68) §	7 (15.91) *	10 (22.73)	15 (68.18)
**Stratified by palpability of the mass**						
**Palpable**						
Current scenario	36 (50.70)	32 (45.07)	35 (49.30)	25 (50.00)	20 (40.00)	25 (50.00)
Scenario #1	27 (38.03) *	30 (42.25)	33 (55.00)	17 (34.00) *	18 (36.00)	23 (57.50)
Scenario #2	20 (28.17) *	27 (38.03)	30 (60.00)	14 (28.00) *	16 (32.00)	20 (58.82)
**Non-Palpable**						
Current scenario	48 (71.64)	14 (20.90)	19 (28.36)	30 (68.18)	8 (18.18)	14 (31.82)
Scenario #1	28 (41.79) *	8 (11.94) †	13 (31.71)	16 (36.36) *	6 (13.64)	10 (38.46)
Scenario #2	19 (28.36) *	7 (10.45) †	11 (36.67)	12 (27.27) *	4 (9.09)	7 (36.84)

Current scenario: all women with BI-RADS-US (HHUS or ABUS) category 4a underwent biopsy; scenario #1: women with BI-RADS-US category 4a and BI-RADS-MAM category 3, 4, and 5 underwent biopsy; scenario #2: women with BI-RADS-US category 4a and BI-RADS-MAM category 4 and 5 underwent biopsy. * *p* < 0.05 for the unnecessary biopsy rate of two new scenarios vs. the current scenario with McNemar’s χ^2^ test. † *p* < 0.05 for the IC detection rate of two new scenarios vs. the current scenario with McNemar’s χ^2^ test. § *p* < 0.05 for the malignancy rate of biopsy of two new scenarios vs. the current scenario with Chi-square test. Abbreviations: ABUS: automated breast ultrasound; BI-RADS: Breast Imaging Reporting and Data System; IC: invasive cancer; HHUS: handheld ultrasound; MAM: mammography.

## Data Availability

The dataset analyzed during the current study is available from the corresponding author upon reasonable request.
